# PHluorin-conjugated secondary nanobodies as a tool for measuring synaptic vesicle exocytosis and endocytosis

**DOI:** 10.1038/s41598-025-92703-4

**Published:** 2025-03-24

**Authors:** Svilen V. Georgiev, Silvio O. Rizzoli

**Affiliations:** Institute for Neuro- and Sensory Physiology and Biostructural Imaging of Neurodegeneration (BIN) Center, University Medical Center Göttingen, 37073 Göttingen, Germany

**Keywords:** Synapse, Vesicle dynamics, PHluorin, Cellular imaging, Cell biology, Neuroscience

## Abstract

Neuronal communication relies on synaptic vesicle recycling, which has long been investigated by live imaging approaches. Synapto-pHluorins, genetically encoded reporters that incorporate a pH-sensitive variant of GFP within the lumen of the synaptic vesicle, have been especially popular. However, they require genetic manipulation, implying that a tool combining their excellent reporter properties with the ease of use of classical immunolabeling would be desirable. We introduce this tool here, relying on primary antibodies against the luminal domain of synaptotagmin 1, decorated with secondary single-domain antibodies (nanobodies) carrying a pHluorin moiety. The application of the antibodies and nanobodies to cultured neurons results in labeling their recycling vesicles, without the need for any additional manipulations. The labeled vesicles respond to stimulation, in the expected fashion, and the pHluorin signals enable the quantification of both exo- and endocytosis. We conclude that pHluorin-conjugated secondary nanobodies are a convenient tool for the analysis of vesicle recycling.

## Introduction

The accurate release of neurotransmitters is essential for neuronal communication, a tightly regulated process that is composed of several mechanisms, including the exo- and endocytosis of transmitter-loaded vesicles. The vesicles are typically categorized into three distinct pools, the readily releasable, recycling and reserve vesicles^1^, with the former participating primarily in exo- and endocytosis, while the latter is less responsive to synaptic activity. The dynamics of these vesicles, collectively known as the synaptic vesicle cycle, have long been studied by a plethora of techniques, and especially by the use of fluorescent probes^2^. Among the most widely employed such tools are the FM dyes, lipophilic fluorophores that are loaded into synaptic vesicles during endocytosis^3,4^, and the pHluorins, genetically-encoded fluorescent proteins fused to the luminal domains of synaptic vesicle proteins, which are quenched by the acidic pH of the vesicles, and thereby report both exo- and endocytosis^5–7^.

Despite their substantial contributions to the field, these tools are not without inherent limitations. FM dyes, for instance, have broad excitation and emission spectra, which can be disadvantageous for multi-color imaging experiments, where spectral overlap must be minimized. Additionally, FM dyes exhibit relatively low brightness and photostability, characteristics that are critical for the accurate live imaging of the synaptic vesicle cycle^8^. The use of FM dyes also necessitates prolonged washing steps, to remove the surface-bound molecules, and thereby reveal the ones trapped in endocytosed vesicles, and to enable exocytosis measurements, in which the FM dye loss from vesicles is measured. The long washing steps may result in the unintended loss of vesicular labeling, due to spontaneous exocytosis. Moreover, FM dyes can interact with various receptor types and potentially influence vesicle exocytosis, thereby confounding experimental results^9–12^. Finally, the FM dyes need to be introduced into the vesicles by a first round of stimulation, which may influence all subsequent experimental steps.

In contrast, the genetically encoded pHluorins are packaged into vesicles by the secretory system of the neurons, and thus remove the problem of dye uptake. Since the development of the first pH-sensitive GFP fused to synaptobrevin 2^7^, researchers have successfully engineered various other pHluorin chimeras, using synaptotagmin^13^, vesicular glutamate transporter 1 (vGlut1)^14^, synaptophysin^15^, or the vesicular GABA transporter (vGAT)^16^. The diversity of the pH-reporters has also advanced through the introduction of red-shifted variants, such as mOrange2, mCherry, and mScarlett^17–19^, broadening the range of fluorescent markers available for live imaging studies. Overall, pH-based reporters offer a broader dynamic range for imaging, with excellent sensitivity, but require the exogenous introduction of genetic material and subsequent expression in neurons, which complicates experimental procedures.

A third category of vesicle-labeling tools is antibodies against the luminal domain of synaptotagmin1 (Syt1), which have been used for more than three decades^20,21^. These antibodies are added to the neurons for tens of minutes or hours, and are internalized during spontaneous exo- and endocytosis events, resulting in abundant synaptic vesicle labeling. As they specifically label synaptic vesicles, unlike the FM dyes, they do not require extensive washing steps, and do not require harsh stimulation paradigms. The latter are needed for FM dyes, since long incubations, under spontaneous culture activity conditions, would also enable the labeling of other types of vesicles and/or endosomes with the dyes, which would confuse the analyses. No such labeling is observed with the Syt1 antibodies, under normal labeling conditions^19^. However, the antibodies cannot be released by the recycling vesicles, implying that they are unable to report exocytosis. To address this problem, they have been conjugated to chemical pH-sensitive fluorophores that detect the acidic environment within vesicles, thereby mimicking the functionality of pHluorins^22^. Their use remains limited, however, especially as CypHer5E, the most commonly used fluorophore, is easily bleached^19,22,23^.

Nonetheless, this approach suggests an optimal use for the Syt1 antibodies. If they could be coupled to pHluorins, they would report exo- and endocytosis in the same fashion as these genetically encoded reporters, without any need for genetic manipulations. We report here this solution, in the form of a tool comprised of an antibody against the luminal domain of Syt1, decorated with a pHluorin-conjugated secondary nanobody. A brief incubation period of these two components forms a stable complex^24,25^, allowing for easy and straightforward labeling of synaptic vesicles. This tool, termed 2^nd^pHluorin integrates the advantages of both anti-Syt1 antibodies and pHluorin-tagged proteins, enabling the analysis of vesicle exocytosis, endocytosis and recycling. This approach adds a reliable, complementary assay to the existing repertoire of methods for imaging synaptic vesicle dynamics.

## Materials and methods

### Animals

All used animals were handled according to the regulations of the University of Göttingen and of the local authorities, the State of Lower Saxony (Landesamt für Verbraucherschutz, LAVES, Braunschweig, Germany). All animal experiments were approved by the local authority, the Lower Saxony State Office for Consumer Protection and Food Safety (Niedersächsisches Landesamt für Verbraucherschutz und Lebensmittelsicherheit) and carried in accordance with the European Communities Council Directive (2010/63/EU). This study was conducted in accordance with ARRIVE guidelines.

## Preparation of primary rat dissociated hippocampal cultures

Newborn rats (Rattus norvegicus, wild-type, Wistar), obtained from previously purchased animals (#RN-WI-F, Janvier-Labs, France) were used to prepare dissociated primary hippocampal cultures, as previously described^26,27^. Shortly, after decapitation of the newborn rats, the hippocampi were dissected in Hank’s Buffered Salt Solution (HBSS, 5 mM KCl, 6 mM glucose, 140 mM NaCl, 4 mM, NaHCO3, 0.4 mM KH2PO4 and 0.3 mM Na2HPO4). Then the tissues were incubated for 60 min in enzyme solution (Dulbecco’s Modified Eagle Medium (DMEM, #D5671, Sigma-Aldrich, Germany), containing 0.5 mg/mL cysteine, 2.5 U/mL papain, 50mM EDTA, 100 mM CaCl2, and saturated with carbogen for 10 min). Subsequently, the dissected hippocampi were incubated for 15 min in a deactivating solution (DMEM containing 0.2 mg/mL trypsin inhibitor, 0.2 mg/mL bovine serum albumin (BSA) and 5% fetal calf serum). Then the cells were triturated and seeded on circular glass coverslips with a diameter of 18 mm at a density of approximately 80,000 cells per coverslip. Prior to seeding, all coverslips underwent treatment with nitric acid, sterilization, and coating overnight (ON) with 1 mg/mL poly-L-lysine. The cells were allowed to adhere to the coverslips for 1–4 h at 37 °C in plating medium (DMEM containing 3.3 mM glucose, 2 mM glutamine, and 10% horse serum). The plating medium was then replaced with a Neurobasal-A medium (Life Technologies, Carlsbad, CA, USA) containing 2% B27 supplement (Gibco, Thermo Fisher Scientific, USA), 1% GlutaMax (Gibco, Thermo Fisher Scientific, USA), and 0.2% penicillin/streptomycin mixture (Biozym Scientific, Germany). Before use, the cultures were maintained in a cell incubator at 37 °C, and 5% CO2 for 12–14 days. Percentages represent volume/volume.

## Production of the synaptic vesicle labeling tool

To generate our specialized synaptic vesicle labeling tool, two key components were utilized: a mouse anti-Syt1 antibody (Cat# 105 311, Synaptic Systems, Göttingen, Germany) and a single-domain camelid antibody (custom made, available upon demand, NanoTag Biotechnologies, Göttingen, Germany). The core sequence of the single-domain camelid antibody was conjugated to the C-terminal pHluorin via a hydrophilic 47-amino acid linker, which included 9 amino acids derived from the original hinge region. This linker was followed by a spacer region containing a TEV protease recognition site (13 amino acids) and a triple FLAG tag (25 amino acids).

## Labeling

Prior labeling, the primary mouse anti-Syt1 antibody at a dilution of 1:500 and the secondary, custom made single-domain camelid antibody with concentration 1 mg/ml and dilution of 1:250, were preincubated in cell culture medium (constituting 10% of the final volume for labeling) for 40 min at room temperature (RT). This pre-incubation step was critical for ensuring the formation of a stable complex between the primary antibody and the secondary single-domain camelid antibody. The volume of the cell culture medium solution, containing our labeling tool was then increased to the final volume needed for the labeling procedure (300 µl per coverslip). After a brief vortexing, 300 µl of labeling solution was pipetted to the wells of a new 12 well plate (Cat# 7696791, TheGeyer, Renningen, Germany). The coverslips were then transferred to the well plate. Subsequently, the neuronal cultures were incubated for 90 min at 37 °C. After the incubation period, the neuronal cultures were washed 3 times in pre-heated Tyrode’s solution (containing 5 mM KCl, 30 mM glucose, 2 mM CaCl2, 124 mM NaCl, 1 mM MgCl2 and 25 mM HEPES, pH 7.4) and returned to their initial well plate, containing their own conditioned media. After an additional period of incubation (60 min), the cells were ready for imaging.

## Transfection procedure

The transfection of neuronal cultures was performed using the Lipofectamine 2000 kit (#11668019, ThermoFisher Scientific, Germany) following standard protocols. Briefly, neuronal cultures were pre-incubated for 25–30 min in 400 µl of pre-heated DMEM (#D5671, Sigma-Aldrich, Germany) per well, supplemented with 10 mM MgCl_2_ at pH 7.5 (fresh-DMEM). During the incubation period, a lipofectamine mix, containing 1 µl lipofectamine solution and 24 µl Opti-MEM (#11058-021, Life Technologies Limited, United Kingdom) per well was prepared. This solution was incubated for 5 min at RT and added to another solution of 1 µg of the synaptophysin-mOrange2 (mOr2-sypHy) plasmid, diluted in a total volume of 25 µl Opti-MEM, per well. The final mixture was incubated for additional 15 min at RT and added to the neurons (in total 50 µl per well). After incubating the neurons at 37 °C and 5% CO2 for 20 min, they were washed twice with fresh-DMEM and returned to their original culture medium. The cultures were then maintained at 37 °C and 5% CO2 until the live-imaging experiments were conducted.

### Stimulation

To block recurrent neuronal activity, 50 µM AP5 (Tocris Bioscience, Bristol, UK; Abcam, Cambridge, UK) and 10 µM CNQX (Tocris Bioscience, Bristol, UK; Abcam, Cambridge, UK) and were added to the imaging solution (preheated Tyrode’s buffer). Electrical stimulation of the cells in Fig. 2a and b was carried with field pulses at a frequency of 20 Hz, lasting for 0.4, 10, 30–40 s at 20 mA and in Fig. 2c was performed at a frequency of 5 and 20 Hz for 30 s with a pause of 30 s between stimulations, and followed by NH_4_Cl treatment. In Fig. 3, the following stimulation paradigm was used: a 10 s baseline measurement, followed by 2 s of stimulation at 20 Hz (40 APs). This was followed by a 30 s pause, then an additional 30 s of stimulation at 20 Hz (600 APs). After a 120 s pause, NH_4_Cl was added to the cells. In Fig. 5 the cells were stimulated five consecutive times, with each stimulus lasting for 2 s at 20 Hz, followed by a 30 s pause. In Fig. 6, neurons were live-labeled with 2^nd^pHluorin for 90 min, then washed and incubated for 48 h, 6 h or 90 min. After this incubation, the cells were stimulated (600 APs, at 20 Hz). At the time of stimulation, the neurons in all conditions were at the same age (DIV14). The stimulation was followed by a NH_4_Cl addition. In Fig. 7, the neurons were stimulated with 1, 5, 10, 20 or 40 APs. All stimulation experiments were conducted with A310 Accupulser Stimulator and 385 Stimulus Isolator (both, World Precision Instruments, Sarasota, FL, USA), and with the help of a platinum custom-made plate field stimulator (with 8 mm distance between the plates).

## Live-Imaging

The coverslips were mounted on a custom-made live-imaging chamber, and 400 µl of pre-heated imaging solution was added (Tyrode’s solution supplemented with the aforementioned drugs). The neurons were then imaged with an inverted Nikon Ti microscope, equipped with Plan Apochromat 60 × 1.4NA oil objective (Nikon Corporation, Chiyoda, Tokyo, Japan), a cage incubator system (OKOlab, Ottaviano, Italy), an Andor iXON 897 emCCCD Camera (Oxford Instruments, Andor), with a pixel size of 16 × 16 μm, and Nikon D-LH Halogen 12 V 100 W Light Lamp House. A temperature of 37 °C was maintained throughout the live-imaging procedure. The illumination and imaging frequency were as follows: 200 ms illumination and 1.7 frames per second (fps) imaging frequency for experiments in Figs. 2 and 200 ms illumination in both channels and 0.55 fps imaging frequency for experiments in Fig. 3.

### Data analysis

The resulting movies were analyzed using custom routines developed in Matlab (The Mathworks Inc., Natick MA, USA; version R2023b). Initially, the frames were drift-corrected to account of any spatial displacement, and were subsequently summed, to generate an overall image with a signal-to-noise ratio superior to that of the individual frames. Synapse coordinates and areas were automatically identified in the summed images using a bandpass filtering procedure^28^, followed by thresholding with an empirically-determined threshold. For each frame, the fluorescence intensity signal within the identified synapse areas was measured, corrected for background signal, and analyzed to assess the changes induced by stimulation. These changes were quantified as the fractional change in intensity relative to the baseline.

For the vesicle pool analysis (Fig. 2c) we measured the peak signals during two rounds of stimulation, first at 5 Hz (30 s), and then at 20 Hz (30 s), and then expressed them as fraction of the respective signals obtained after NH_4_Cl dequenching. Synapses providing negative signals for any of the three measurements were removed from the analysis, as they were considered to not respond properly to stimulation.

### Statistical analysis and visualization

For statistical analysis Matlab (The Mathworks Inc., Natick MA, USA; version R2023b) and GraphPad Prism 14.5 (GraphPad Software LLC) were used. For creation of the graphs Matlab and CorelDraw (Version 24.5.0.731) were used.

## Results

### 2^nd^pHluorin successfully reports synaptic vesicle exocytosis

**Fig. 1:**
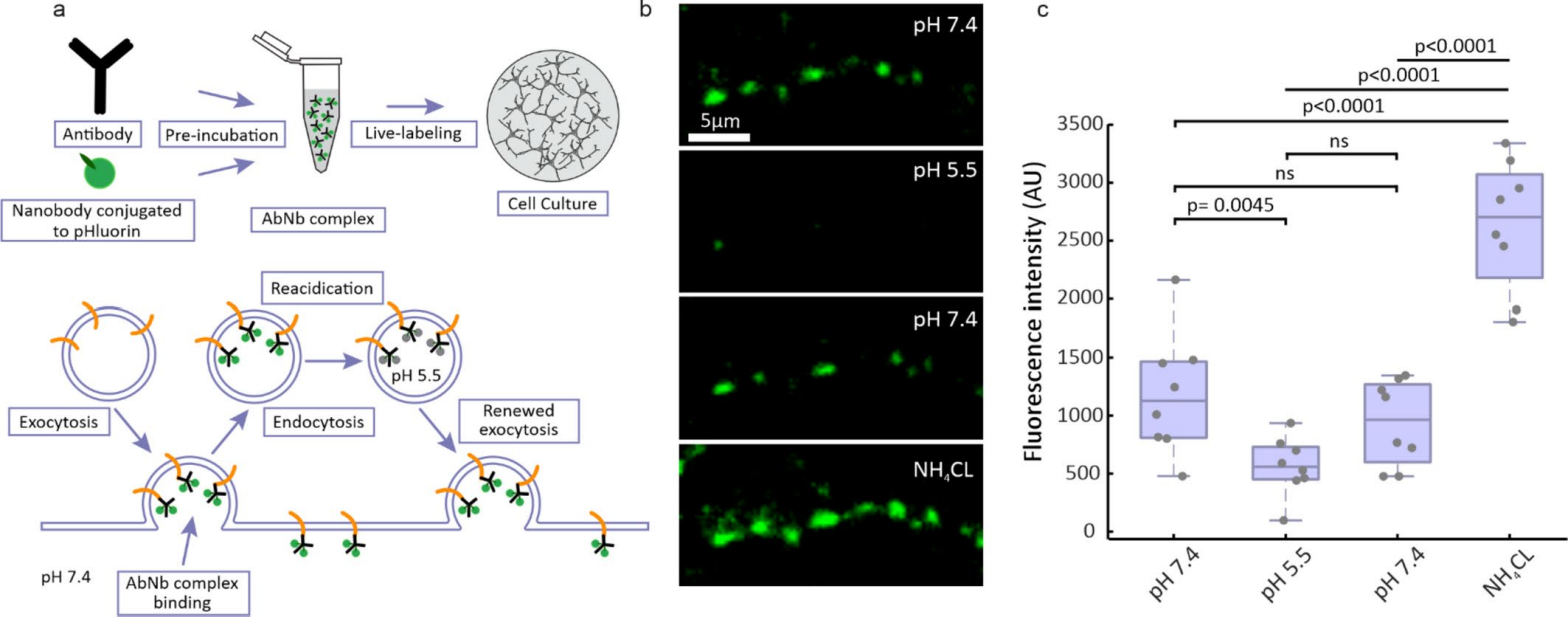
Experimental setup for recording synaptic activity. (**a)** Schematic of the synaptic labeling procedure, using a primary antibody and a secondary single-domain camelid antibody (nanobody) conjugated to pHluorin. The antibody-nanobody complex (AbNb) is taken up by vesicles during a live-labeling period of 90 min, thereby labeling the entire recycling pool. The pHluorin moiety is then able to react to exo- and endocytosis, as it is quenched in the acidic interior of the synaptic vesicle, but it is allowed to fluoresce at neutral pH. **(b)** Representative images of synaptic boutons subjected to different pH conditions, with 100 mM NH_4_Cl applied at the end, to induce alkalinization. **(c)** Quantification of fluorescence intensity across synaptic boutons (*n* = 280), over 8 independent experiments (*N* = 8). Each black circle represents the mean value per experiment. Data are presented as a boxplot showing the median and quartiles, while whiskers represent the full data range. Statistical analysis was performed using One-Way ANOVA followed by Tukey’s LSD test.

To validate the pH sensitivity of 2^nd^pHluorin, we conducted a classical experiment in which we manipulated the pH of the cell culture buffers and monitored the resulting changes in fluorescence intensity at synaptic boutons^29^. We labeled neurons live by applying 2^nd^pHluorin to the neuronal cultures over a 90-minute incubation. The uptake of our tool occurred through spontaneous exo- and endocytosis, under conditions of spontaneous culture activity, as is typically performed for these antibodies^19^. Once endocytosed, the acidic lumen of the vesicles quenches the pH-sensitive reporter (Fig. 1a). Upon re-exocytosis, the pH-sensitive reporter is exposed to the neutral extracellular pH, allowing it to fluoresce upon illumination. As shown in Fig. 1b and c, baseline fluorescence intensity, which reveals Syt1 molecules found on the neuronal surface (known as surface pool), significantly decreased when the extracellular pH was lowered to 5.5. Upon replacement of the acidic solution with Tyrode’s buffer at pH 7.4, the fluorescence intensity nearly returned to its initial level. We then perfused the cells with a buffer containing 100 mM NH_4_Cl. This results in the diffusion of ammonia across cell membranes, leading to the elevation of cytosolic and organelle pH^30^. As expected, a substantial rise in fluorescence intensity was observed. These results demonstrate that 2^nd^pHluorin effectively labels synapses, and is capable of sensing changes in the pH at the synapse.

### 2^nd^pHluorin senses exo- and endocytosis upon stimulation

**Fig. 2:**
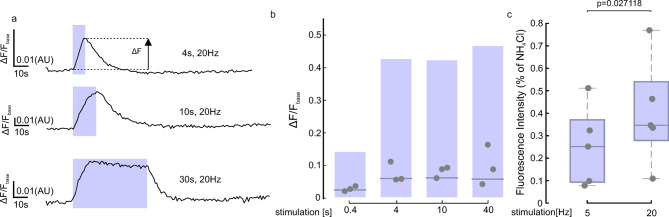
2^nd^pHluorin successfully responds to various stimulation paradigms. **(a)** Traces showing changes in fluorescence intensity (expressed as fluorescence normalized to the baseline signal) from 3 different experimental paradigms, in which synapses were stimulated for 4, 10–30 s, at 20 Hz. **(b)** Flying bars showing the changes in fluorescence during the peak response (as indicated in **a**) to stimulations for 0.4, 4, 10 and 40 s at 20 Hz. The line indicates the median value, the edges of the box the minimum and maximum values. *N* = 3 experiments per condition with 251, 244, 441, and 390 boutons analyzed per respective conditions. **(c)** Boxplots showing the changes in fluorescence during the peak response to stimulations at 5 Hz and 20 Hz (for 30 s), normalized to the fluorescence intensity changes after NH_4_Cl treatment. *N* = 5 independent experiments. Statistical analysis was performed using a two-tailed paired t-test.

We next tested the performance of 2^nd^pHluorin under different electrical stimulation paradigms. To prevent recurrent neuronal activity, we supplemented our imaging solution with d, l-2-amino-5-phosphonovaleric acid (AP5) and 6-cyano-7-nitroquinoxaline-2,3-dione (CNQX). We employed various stimulation protocols, ranging from 8 to 800 action potentials (APs) at 20 Hz. Across all experiments, we observed significant fluorescence intensity changes relative to baseline fluorescence (Fig. 2a and b). The application of 2^nd^pHluorin enabled us to detect exocytosis events from the readily releasable pool of synaptic vesicles, which are immediately available for release upon short stimulations (8, 80 APs)^31^. Additionally, we detected exocytosis from the recycling pool, which is mobilized following more sustained stimulation (200, 600, 800 APs)^19,32^. Moreover, our procedure enabled us to label vesicles belonging to the reserve pool, as indicated by comparing the fluorescence intensity changes from baseline after stimulations with 150 APs and 600 APs, normalized to the change of fluorescence intensity signal, following NH_4_Cl application (Fig. 2c). The 600 APs stimulus is suggested to release the entire recycling pool^19,31,33^, and it appears to cause the exocytosis of only ~30–40% of all pHluorin-labeled vesicles, which is consistent with values observed in studies investigating the reserve pool and using conventional pHluorin reporters or other tools^19,31^. These findings demonstrate that 2^nd^pHluorin is a sensitive tool that enables the analysis of exocytosis and recycling of different synaptic vesicle populations.

### Labeling of different synaptic vesicle pools with 2^nd^pHluorin

**Fig. 3:**
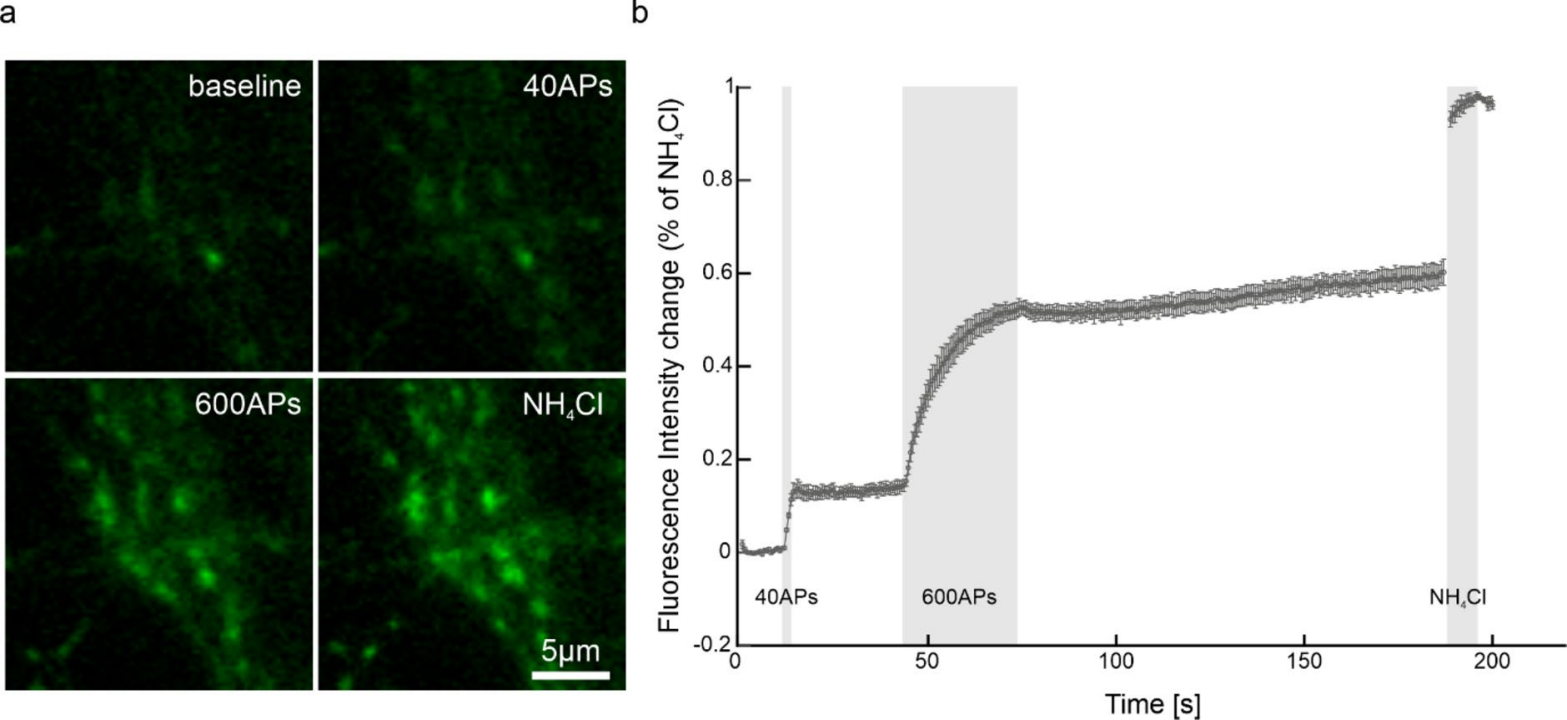
Labeling of different synaptic vesicle pools with 2^nd^pHluorin. (**a)** Images showing the fluorescence intensity of synaptic boutons upon different stimulation paradigms and NH_4_Cl application. The experiment was performed in bafilomycin-containing buffer (1.5 µM). (**b)** Stimulation curve showing the mean fluorescence of 1817 synaptic boutons across 5 independent experiments (*N* = 5). The fluorescence increase was expressed as a fraction of the signal observed upon NH_4_Cl application (i.e., as percentage of all labeled vesicles). The error bars represent s.e.m.

Next, we assessed the ability of 2^nd^pHluorin to label synaptic vesicles from the readily releasable pool (RRP), the recycling pool, and the reserve pool. To achieve this, we designed an experimental paradigm consisting of a brief 2 s stimulation at 20 Hz (40 APs), designed to release the RRP, followed by a 30 s pause, and a subsequent 30 s stimulation at 20 Hz (600 APs), which should release the recycling pool. After an additional pause of 120 s, NH₄Cl was applied to reveal all other vesicles. We conducted these experiments in a buffer containing 1.5 µM bafilomycin, a V-ATPase inhibitor that prevents synaptic vesicle re-acidification after endocytosis, keeping them in an alkaline state^34,35^ (Fig. 3a, b). Approximately 40% of the labeled vesicles were part of the reserve pool, consistent with previous literature referring to the labeling of hippocampal vesicle pools using antibodies^19^. These findings demonstrate that 2^nd^pHluorin is a sensitive tool for analyzing exocytosis and the recycling dynamics of distinct synaptic vesicle populations.

### Comparative analysis of 2^nd^pHluorin and mOr2-sypHy

**Fig. 4:**
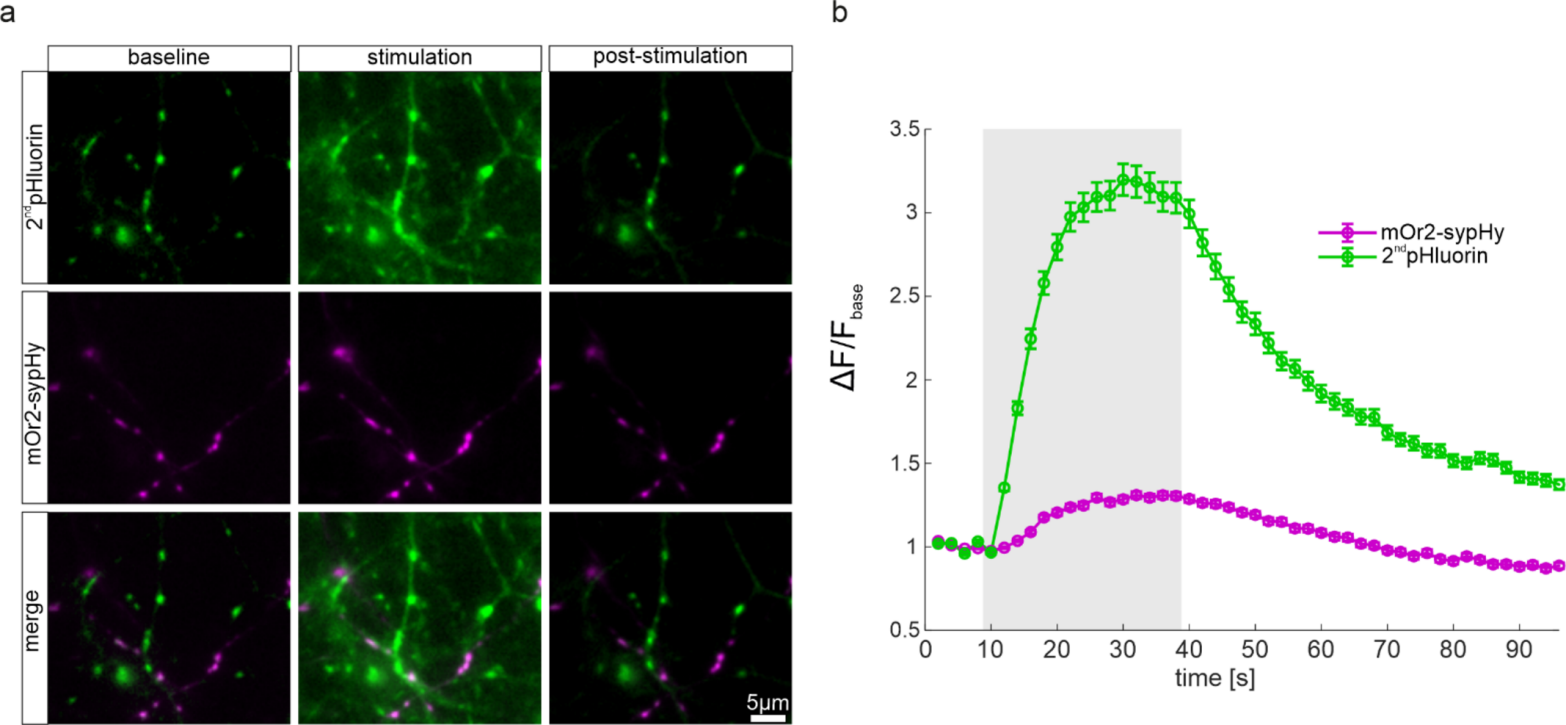
Comparison between 2^nd^pHluorin and Synaptophysin-mOrange2. (**a)** Representative images of synaptic boutons from hippocampal neurons transfected with mOr2-sypHy and stained with 2^nd^pHluorin before, during, and after application of a stimulus of 600 APs at 20 Hz. (**b)** Representative signal curves from 2^nd^pHluorin (green) and mOr2-sypHy (magenta) in response to 600 APs at 20 Hz (*N* = 3 experiment). The error bars represent s.e.m.

To assess the efficacy of 2^nd^pHluorin in labeling synaptic boutons and studying vesicle recycling, relative to existing methods, we conducted dual-color live imaging experiments on hippocampal neuronal cultures transfected with synaptophysin-mOrange2 (mOr2-sypHy). The mOr2-sypHy construct is a modified version of the original sypHy probe, wherein the superecliptic GFP is replaced by the pH-sensitive mOrange2 fluorescent protein^19,36^. Due to the transient plasmid expression used for mOr2-sypHy, we observed relatively low transfection efficiency, resulting in only a few labeled cells per coverslip. In contrast, our 2^nd^pHluorin tool demonstrated substantially higher labeling efficiency across the neuronal cultures, as it is expected to label all active synapses. Nevertheless, we successfully labeled functional synaptic boutons expressing mOr2-sypHy with 2^nd^pHluorin, as illustrated in Fig. 4a. We quantified the fluorescence intensity changes relative to baseline (ΔF/Fbase) in these dual-labeled boutons, upon stimulation with 600 APs. As depicted in Fig. 4b, 2^nd^pHluorin exhibited a significantly greater dynamic range, when compared to mOr2-sypHy, indicating a more robust detection of synaptic vesicle exocytosis events. This is in line with previous results^19^, which indicate that the pH-dependent changes in mOr2 fluorescence are lower than those undertaken by the GFP-based pHluorin. Nonetheless, the kinetics observed with the two fluorophores are similar, indicating that the 2^nd^pHluorin provides qualitatively identical results to genetically-encoded detectors.

### Reliability and limitations of 2^nd^pHluorin

**Fig. 5:**
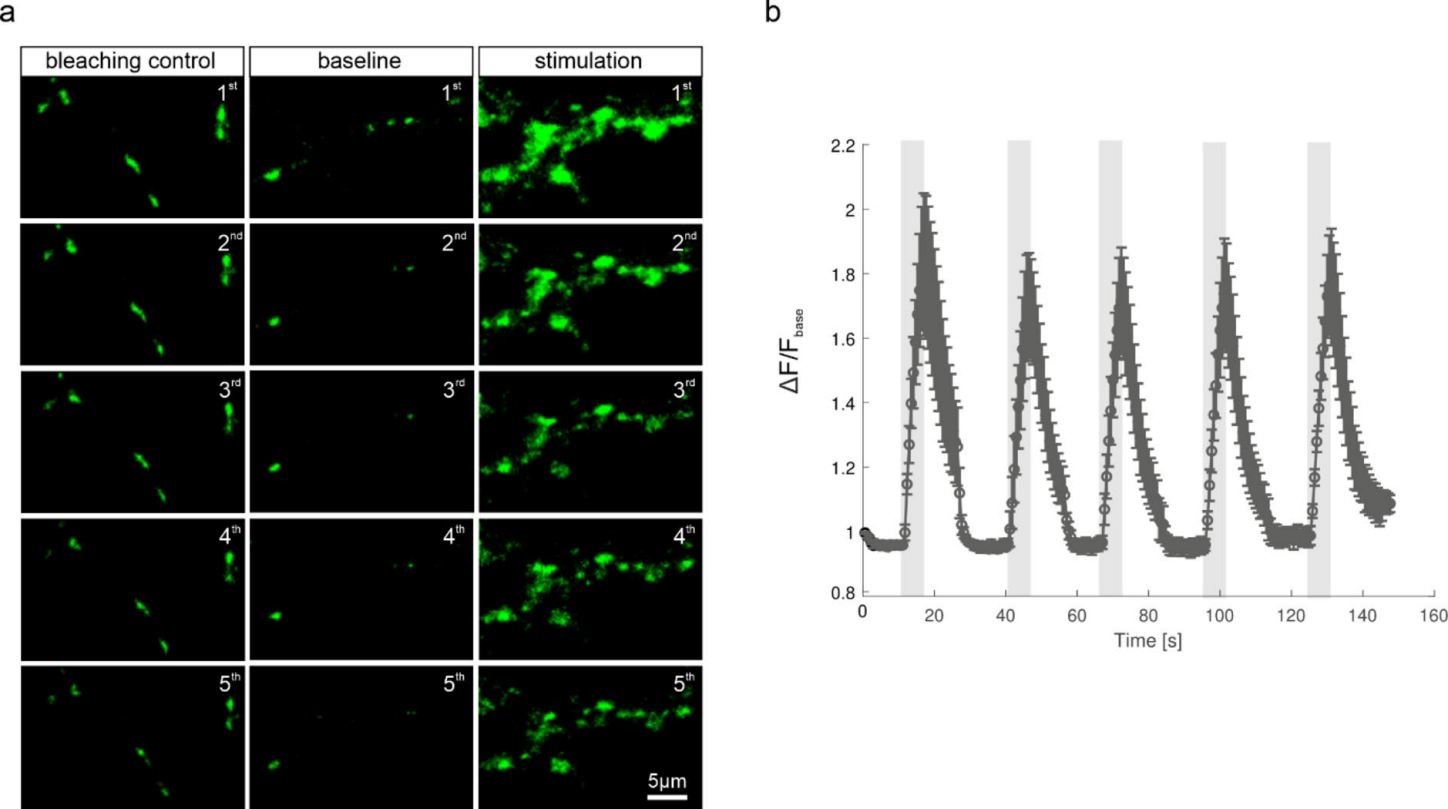
Repeated stimulation of the same synaptic boutons labeled with 2^nd^pHluorin. **(a)** Repeated imaging examples. The leftmost column shows a control for the signal lost through photobleaching, imaged in exactly the same fashion as the experimental conditions, but without stimulation. The middle column shows the baseline fluorescence intensity prior to stimulation, while the rightmost column shows the change in the fluorescence intensity upon stimulation with 100 APs. **(b)** Stimulation curve, showing the mean fluorescence intensity values of 996 synaptic boutons, across 4 independent experiments (*N* = 4). The curve was normalized to the first time frame and was corrected for photobleaching. The error bars represent s.e.m.

To assess whether 2^nd^pHluorin remains within vesicles over multiple stimulation rounds, we imaged neurons subjected to five consecutive trains of 100 APs, at 20 Hz (Fig. 5). We detected fluorescence intensity changes across all five stimulations, with no significant differences in response amplitude.

**Fig. 6:**
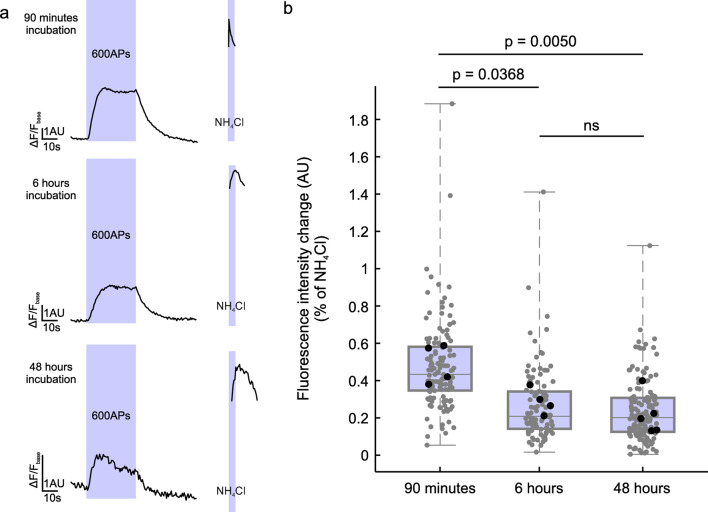
Stimulation of synaptic boutons after different incubation periods. **(a)** Average fluorescence signal from neurons labeled with 2^nd^pHluorin and then subjected to incubation times of 90 min, 6 and 48 h. The traces show fluorescence intensity changes upon electrical stimulation with 600 APs and upon addition of NH_4_Cl. **(b)** Quantification of peak fluorescence intensity, as a percentage of fluorescence intensity change after NH_4_Cl application. The following number of synaptic boutons were analyzed: 90 min 129 synapses, 6 h 104 synapses, 48 h 141 synapses, from 5 experiments in the 48 h condition and 4 experiments in the 90 min and 6 h conditions. Each black circle represents the mean value per experiment, while each grey circle represents a synaptic bouton. Data are presented as a box plot showing the median and quartiles, with whiskers representing the full data range. Normality of the data was tested with Shapiro-Wilk Test. Statistical analysis was performed using one-way ANOVA, followed by Tukey’s multiple comparisons test.

**Fig. 7:**
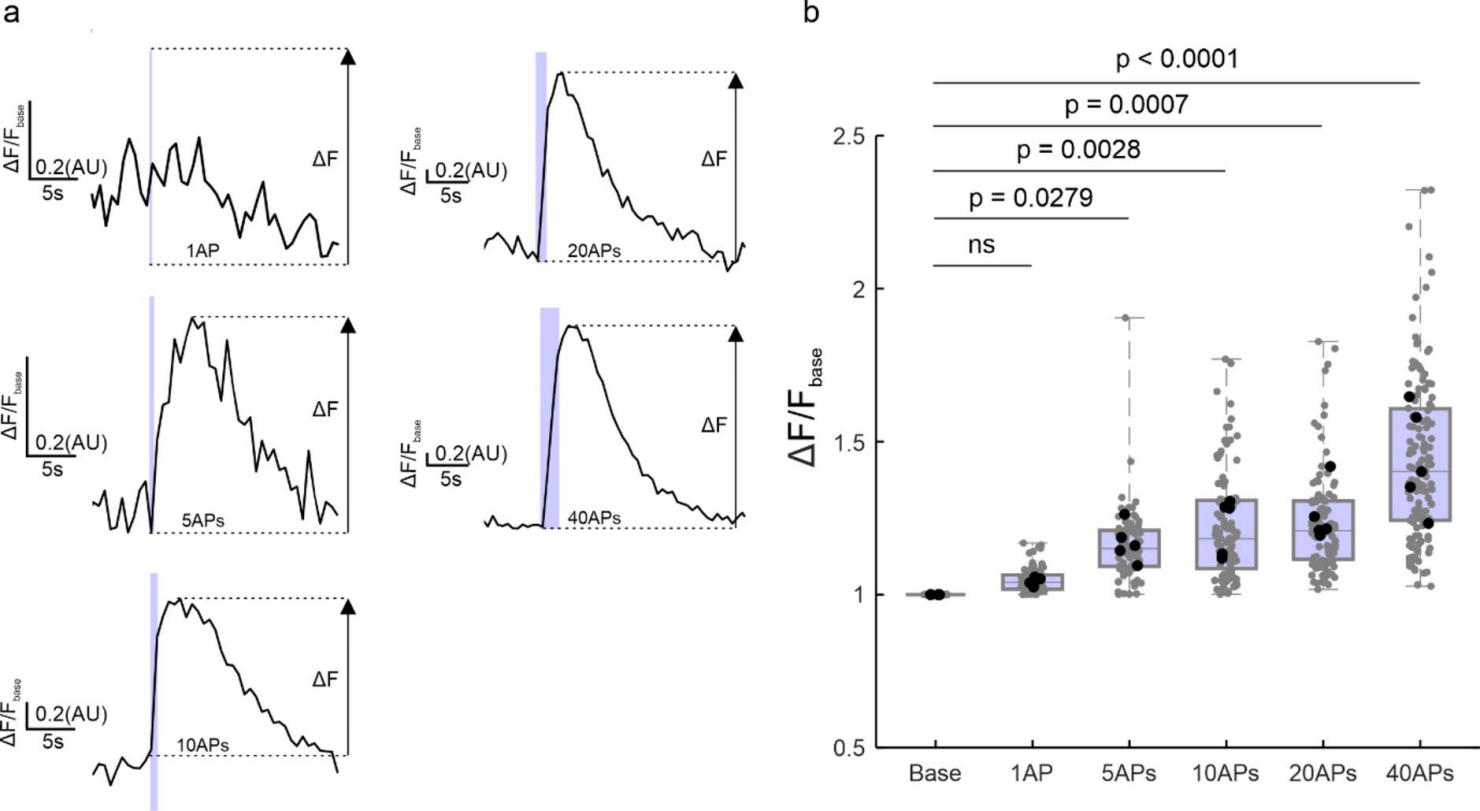
Sensitivity of 2^nd^pHluorin. (**a)**. Traces showing fluorescence intensity changes, (expressed as fluorescence normalized to the baseline signal) upon stimulations with 1, 5, 10, 20 or 40 APs. (**b)** Quantification of fluorescence intensity across synaptic boutons. An arbitrary value of 1 was assigned for the baseline (‘Base’), and this value was compared to all stimulation paradigms. The following number of synaptic boutons per condition were analyzed: 1 AP = 79 synapses, 5 APs = 82 synapses, 10 APs = 108, 20 APs = 112, 40 APs = 135, *N* = 5 independent experiments. Each black circle represents the mean value per experiment. Each grey circle represents a synaptic bouton. Data are presented as a box plot showing the median and quartiles, while whiskers represent the full data range. Statistical analysis was performed using one-way ANOVA, followed by Tukey’s multiple comparisons test.

To test the behavior of the 2^nd^pHluorin over longer time periods, we incubated the labeled cultures for 6–48 h before subjecting them to a standard stimulation paradigm of 600 APs (Fig. 6). The proportion of the vesicles that responded to stimulation was reduced by incubation, presumably through the sorting of labeled synaptic vesicles to the inactive, reserve pool^19^. Next, we aimed to determine the lower detection limit of our experimental setup. Synaptic boutons were stimulated with 1, 5, 10, 20, and 40 APs (Fig. 7). While the response to 1 AP was indistinguishable from baseline, all other stimuli produced a detectable increase in fluorescence intensity.

## Discussion

In this study, we demonstrated that a tool composed of primary antibodies against Syt1 and secondary nanobodies conjugated to pHluorin (2^nd^pHluorin) exhibits robust performance in monitoring synaptic vesicle dynamics in primary hippocampal rat neuronal cultures. It labels synaptic boutons and detects pH-changes in their vesicles, characteristics that render it a reliable pH reporter. 2^nd^pHluorin also responds to a wide range of stimulation paradigms, from 5 APs to 800 APs. In addition, 2^nd^pHluorin is able to label different synaptic vesicle pools, including the readily releasable pool (RRP), the recycling pool and the reserve pool. Our tool is suitable for experimental conditions requiring longer incubation periods as well as studies involving repeated stimulation at the same synaptic boutons. 2^nd^pHluorin offers superior labeling efficiency improved signal to noise ratio, compared to reporters as mOrange2. It also offers. These features make 2^nd^pHluorin a convenient tool for studying synaptic vesicle recycling.

While our tool shows promising performance in studying synaptic vesicle dynamics, several challenges remain to be addressed. Although it avoids the need of extensive washing steps, as in the case of FM dyes, it still requires a pre-incubation period, during which the antibody-nanobody complex is formed and then taken up by the vesicles. This step typically takes approximately 130 min, and requires primary antibody to secondary nanobody ratios of at least 1:2, to ensure that all binding sites on the primary antibody are occupied by the pH-conjugated nanobody^25^. Nevertheless, this process is faster than conventional transfection techniques, as lipofectamine or calcium phosphate transfection, and, crucially, it also bypasses the need for genetic manipulation of neurons. A possible solution to this challenge would be the direct conjugation of the primary antibody to a pH-reporter. This has already been employed with tools like CypHer5E fused to Syt1^22^, a pH reporter that operates in an opposite manner to most pHluorins, since it is quenched at neutral pH, and is activated by the acidic environment inside the vesicles. A promising next step would involve the development of nanobodies against Syt1, that are directly conjugated to pHluorin. Syt1 nanobodies against the cytosolic parts of Syt1 already exist, and have been successfully employed in neurophysiological studies^37^, but nanobodies against its luminal domain still need to be characterized.

Another significant challenge when using 2^nd^pHluorin is the limited fraction of synaptic vesicles labeled during the pre-incubation procedure. This limitation arises from the fact that 2^nd^pHluorin specifically labels the luminal epitopes of Syt1 that are exposed to the extracellular milieu during spontaneous exo- and endocytosed events, thereby exclusively targeting vesicles that undergo active exo- and endocytosis. The reserve pool of vesicles, which remains inactive during physiological conditions, only fuses with the plasma membrane following prolonged, non-physiological electrical stimulations, or the application of pharmacological agents of drugs that enhance presynaptic activity^1,38,39^. To label reserve vesicles, the user needs to wait a significant time period after labeling (preferably hours), to ensure that the newly recycled vesicles intermix functionally with the reserve pool^19^. Nevertheless, the incubation time of 60 min, after the 90-minutes labeling procedure, was sufficient to label at least a fraction of the reserve vesicle pool, in our experiments (Figs. 2c and 3).

In conclusion, we believe that our tool is a valuable contribution to the existing methods for studying synaptic vesicle dynamics.

## Data Availability

The datasets used and/or analysed during the current study available from the corresponding author on reasonable request.
